# Inhibitors of DNA Methyltransferases From Natural Sources: A Computational Perspective

**DOI:** 10.3389/fphar.2018.01144

**Published:** 2018-10-10

**Authors:** Fernanda I. Saldívar-González, Alejandro Gómez-García, David E. Chávez-Ponce de León, Norberto Sánchez-Cruz, Javier Ruiz-Rios, B. Angélica Pilón-Jiménez, José L. Medina-Franco

**Affiliations:** Department of Pharmacy, School of Chemistry, National Autonomous University of Mexico, Mexico City, Mexico

**Keywords:** chemical space, chemoinformatics, databases, DNMT inhibitors, drug discovery, molecular modeling, similarity searching, virtual screening

## Abstract

Naturally occurring small molecules include a large variety of natural products from different sources that have confirmed activity against epigenetic targets. In this work we review chemoinformatic, molecular modeling, and other computational approaches that have been used to uncover natural products as inhibitors of DNA methyltransferases, a major family of epigenetic targets with therapeutic interest. Examples of computational approaches surveyed in this work are docking, similarity-based virtual screening, and pharmacophore modeling. It is also discussed the chemoinformatic-guided exploration of the chemical space of naturally occurring compounds as epigenetic modulators which may have significant implications in epigenetic drug discovery and nutriepigenetics.

## Section 1: Introduction

Epigenetics has been defined as a change in phenotype without an underlying change in genotype (Berger et al., 2009). In the 1940s Waddington suggested the term “epigenetics” trying to describe “the interactions of genes with their environment, which brings the phenotype into being” (Waddington, 2012). Alterations in epigenetic modifications have been related to several diseases including cancer, diabetes, neurodegenerative disorders, and immune-mediated diseases (Dueñas-González et al., 2016; Tough et al., 2016; Hwang et al., 2017; Lu et al., 2018). Moreover, epigenetic targets are also attractive for the treatment of antiparasitic infections (Sacconnay et al., 2014).

In epigenetic drug discovery, epigenetic targets have been classified into three main groups (Ganesan, 2018). “Writers” are enzymes that catalyze the addition of a functional group to a protein or nucleic acid; “readers” are macromolecules that function as recognition units that can distinguish a native macromolecule vs. the modified one; and “erasers” that are enzymes that aid in the removal of chemical modifications introduced by the writers. Thus far, several targets from these three major families have reached different stages of drug discovery, ranging from lead discovery, preclinical development, clinical trials and approval. Currently, there are seven compounds approved for clinical use (Ganesan, 2018).

DNA methyltransferases (DNMTs) are a family of “writer” enzymes responsible for DNA methylation that is the addition of a methyl group to the carbon atom number five (C5) of cytosine. As surveyed in this work, since DNA methylation has an essential role for cell differentiation and development, alterations in the function of DNMTs have been associated with cancer (Castillo-Aguilera et al., 2017) and other diseases (Lyko, 2017).

Several natural products have been identified as inhibitors of epigenetic targets including DNMTs. Most of these compounds have been uncovered fortuitously. However, there are recent efforts to screen systematically natural products as DNMT inhibitors. The vastness of the chemical space of natural products led to the hypothesis that many more active compounds could potentially been identified. Indeed, it has been estimated that more than 95% of the biodiversity in nature remains to be explored to identify potential bioactive molecules (Ho et al., 2018).

The aim of this work is to discuss a broad range of computational methods to identify novel inhibitors of DNMTs from natural products. The manuscript also discusses the chemical space of natural products as inhibitors of DNMTs. The manuscript is organized into nine sections. After this introduction, Section 2 reviews briefly the structure of DNMTs including different isoforms. The next section covers major aspects of the function of DNMTs including the mechanism of methylation. Section 4 reviews currently known inhibitors of DNMTs from natural sources including food chemicals. Section 5 discusses the epigenetic relevant chemical space of natural products comparing the chemical space of DNMT inhibitors from natural sources vs. other compounds. The next section reviews computational strategies that are used to identify natural compounds as potential epi-hits or epi-leads targeting DNMTs. Sections 7 and 8 presents Summary conclusions and Perspectives, respectively.

## Section 2: Structure of Dnmts

**FIGURE 1:**
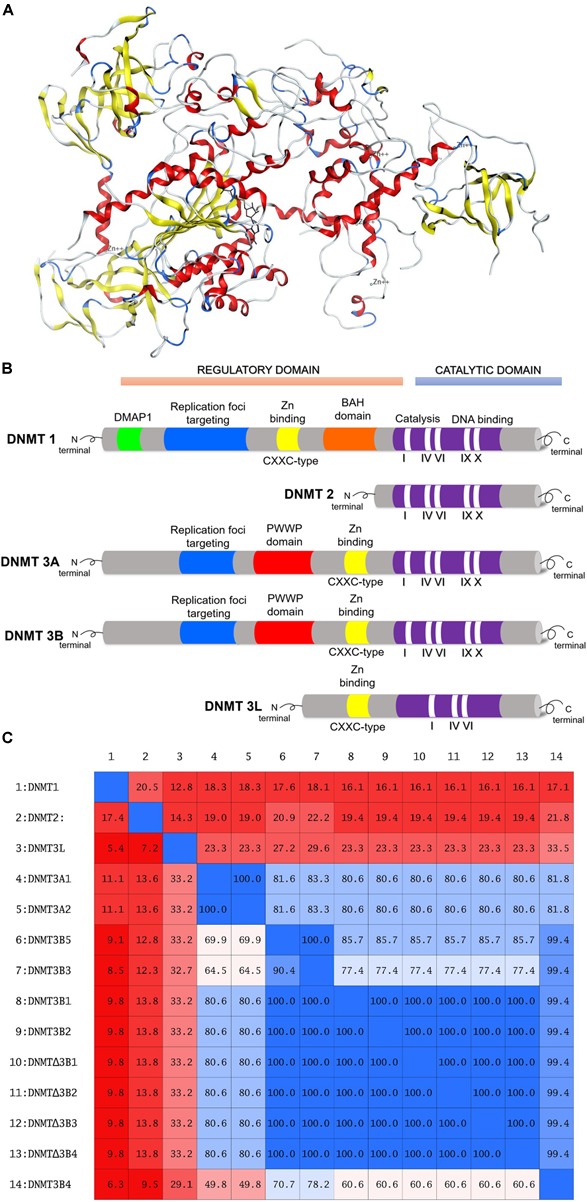
**(A)** Three-dimensional model of DNMT1, amino acid residues 351–1600. Figure rendered from the Protein Data Bank PDB ID: 4WXX. **(B)** Schematic diagram of the structure of human DNMT1, DNMT2, DNMT3A, DNMT3B, and DNMT3L. **(C)** Identity matrix of the catalytic site of 14 DNMTs isoforms. Note that there is a significant difference in the sequence of DNMT1, DNMT2, and DNMT3L.

The human genome encodes DNMT1, DNMT2, DNMT3A, DNMT3B, and DNMT3L. While DNMT1, DNMT3A, and DNMT3B have catalytic activity, DNMT2 and DNMT3L do not (Lyko, 2017). DNMT1 is a maintenance methyltransferase, responsible for duplicating the pattern of DNA methylation during replication. DNMT1 is essential for proper mammalian development and it has been proposed as the most interesting target for experimental cancer therapies (Dueñas-González et al., 2016). DNMT3A and DNMT3B are *de novo* methyltransferases. Human DNMT1 has 1616 amino acids whose structure can be divided into an N-terminal regulatory domain and a C-terminal catalytic domain (Jeltsch, 2002; Jurkowska et al., 2011). The N-terminal domain contains a replication foci-targeting domain, a DNA-binding CXXC domain, and a pair of bromo-adjacent homology domains. The C-terminal catalytic domain has 10 amino acid motifs. The cofactor and substrate binding sites in the C-terminal catalytic domain are comprised of motif I and X and motif IV, VI, and VIII, respectively (Lan et al., 2010). The target recognition domain which is maintained by motif IX and involved in DNA recognition, is not conserved between the DNMT family. **Figure 1A** shows a three-dimensional (3D) model of a DNMT1 (PDB ID: 4WXX) (Zhang et al., 2015). **Figure 1B** shows a schematic diagram of human of DNMT1, 2, 3A, 3B, and L.

### Section 2.1: Isoforms

Two isoforms of DNMT3A have been identified, DNMT3A1 and DNMT3A2. At the N-terminal domain both isoforms have a PWWP (Pro-Trp-Trp-Pro) and an ADD (ATRX-DNMT3-DNMT3L) domains (Jurkowska et al., 2011). The C-terminal domain is identical in the two isoforms (Choi et al., 2011).

There are more than 30 isoforms of DNMT3B, however, only DNMT3B1 and DNMT3B2 are catalytically active (Ostler et al., 2007). Similar to DNMT3A, DNMT3B1, and DNMT3B2 have a PWWP and ADD domains at the N-terminal region (Lyko, 2017). The rest of the isoforms are not catalytically active. Some of these such as DNMT3B3, DNMT3B4, and DNMT3B7 are overexpressed in many tumor cell lines (Gordon et al., 2013). ΔDNMT3B has seven isoforms and lacks 200 amino acids from the N-terminal region of DNMT3B (Wang et al., 2006). ΔDNMT3B1–4 possess catalytic activity whereas ΔDNMT3B5–7 lacks the catalytic domain (Wang et al., 2006). ΔDNMT3B is mainly expressed in non-small cell lung cancer (Wang et al., 2006; Ostler et al., 2007). **Figure 1C** shows the identity matrix of 14 DNMTs isoforms. The identity matrix indicates that the amino acid sequence at the catalytic site of DNMT3A1 and DNMT3A2 isoforms is identical. In the same manner, the amino acid sequence at the C-terminal domain of the catalytically active isoforms DNMT3B1, DNMT3B2, and ΔDNMT3B1–4 are identical. DNMT1, DNMT2, and DNMT3L show a significant difference in the sequence of the catalytic site with respect to the rest of the isoforms. Therefore, it can be anticipated that is possible to identify or design selective inhibitors for these isoforms.

## Section 3: Function and Mechanism of Dnmts

As outlined in Section 2, cytosine-5 DNMTs catalyze the addition of methylation marks to genomic DNA. All DNMTs have a related catalytic mechanism that is featured by the formation of a covalent adduct intermediate between the enzyme and the substrate base. All DNMTs use *S*-adenosyl-L-methionine (SAM) as the methyl group donor (Vilkaitis et al., 2001; Du et al., 2016). DNMT forms a complex with DNA and the cytosine which will be methylated flips out from the DNA (Klimasauskas et al., 1994). A conserved cysteine performs a nucleophilic attack to the six-position of the target cytosine yielding a covalent intermediate. The five-position of the cytosine is activated and conducts a nucleophilic attack on the cofactor SAM to form the 5-methyl covalent adduct and *S*-adenosyl-*L*-homocysteine (SAH). The attack on the six-position is aided by a transient protonation of the cytosine ring at the endocyclic nitrogen atom N3, which can be stabilized by a glutamate and arginine residues. The covalent complex between the methylated base and the DNA is resolved by deprotonation at the five-position to generate the methylated cytosine and the free enzyme.

## Section 4: Known Inhibitors of Dnmts From Natural Sources

Thus far more than 500 compounds have been tested as inhibitors of DNMTs. The structural diversity and coverage in chemical space has been analyzed using chemoinformatic methods (Fernandez-de Gortari and Medina-Franco, 2015). The chemical space of DNMT inhibitors has been compared with inhibitors of other epigenetic targets (Naveja and Medina-Franco, 2018). Furthermore, the structure-activity relationships (SAR) of DNMT inhibitors using the concept of activity landscape has been documented (Naveja and Medina-Franco, 2015).

**FIGURE 2:**
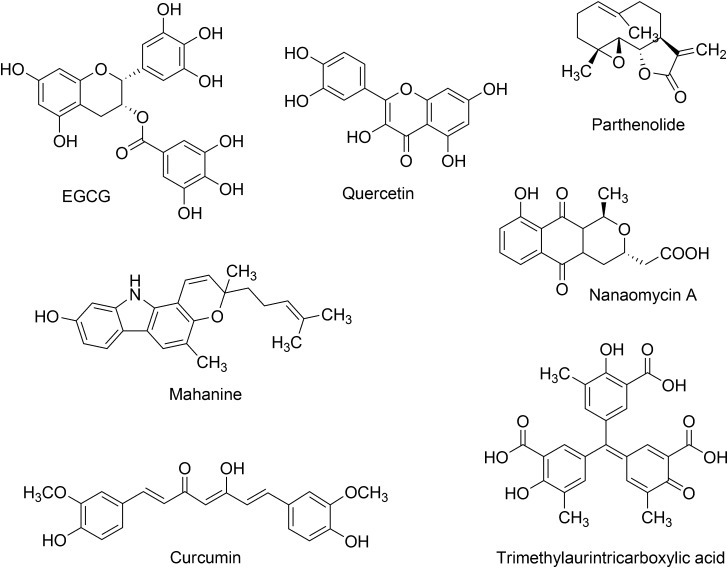
Chemical structures of representative inhibitors of DNMTs from natural sources. These natural products are extensively reviewed by Zwergel et al. (2016).

DNA methyltransferase inhibitors have been obtained from a broad number of different strategies including organic synthesis, virtual, and high-throughput screening (Medina-Franco et al., 2015). Organic synthesis has been employed in several instances for lead optimization (Castellano et al., 2008; Kabro et al., 2013; Davide et al., 2016). Natural products and food chemicals have also been a major source of active compounds. Natural products that are known to act as DNMT inhibitors or demethylating agents have been extensively reviewed by Zwergel et al. (2016). These natural products are of the type polyphenols, flavonoids, anthraquinones, and other classes. Some of the first natural products described were curcumin, (-)-epigallocatechin-3-gallate (EGCG), mahanine, genistein, and quercetin. Other natural products that have described as inhibitors of DNMT or demethylating agents are silibinin, luteolin, kazinol Q, laccaic acid, hypericin, boswellic acid, and lycopene. **Figure 2** shows the chemical structure of representative DNMT inhibitors with emphasis on compounds from natural origin.

The bioactivity profile and potency in enzymatic and/or cell-based assays of these natural products have been discussed in detail by Zwergel et al. (2016). Of note, it will be valuable if all natural products could have been screened under the same conditions. For few natural products the selectivity has been characterized being nanaomycin A an exception (*vide infra*). Indeed, for about eight natural products the IC_50_ has been measured in enzymatic based assays. Despite the fact that the potency of the natural products with DNMTs is not very high in enzymatic-based assays, e.g., IC_50_ between 0.5 and 10 μM, several natural products have shown promising activity in cell-based assays. Notably, natural products have distinct chemical scaffolds that could be used as a starting point in lead optimization efforts. Moreover, quercetin in combination with green tea extract has advanced into phase I clinical trials for the treatment of prostate cancer.

Most of the natural products with demethylating activity or ability to inhibit DNA methyltransferases in enzymatic assays have been identified fortuitously. However, as discussed in this work, there are efforts toward the identification of bioactive demethylating agents using systematic approaches such a virtual screening. Indeed, the natural product nanaomycin A (**Figure 2**) was identified from a virtual screening campaign initially focused on the identification of inhibitors of DNMT1. The quinone-based antibiotic isolated from *Streptomyces* showed antiproliferative effects in three human tumor cell lines, HCT116, A549, and HL60 after 72 h of treatment. Moreover, nanaomycin A showed reduced global methylation levels in all three cell lines when tested at concentrations ranging from 0.5 to 5 μM. Nanaomycin A reactivated the transcription of the RASSF1A tumor suppressor gene inducing its expression up to 18-fold at 5 μM, higher than the reference drug 5-azacytidine (sixfold at 25 μM). In an enzymatic inhibitory assay, nanaomycin A was selective toward DNMT3B with an IC_50_ = 0.50 μM.

### Section 4.1: Natural Products and Food Chemicals

It is remarkable that several natural products are used as dietary sources such as curcumin, caffeic acid and chlorogenic acid found in *Coffea arabica*, genistein found in soybean, quercetin found in fruits, vegetables, and beverages. Of course, there is a large overlap between the chemical space of food chemicals and natural products (Naveja et al., 2018). This has given rise to systematically screen food chemical databases for potential regulators of epigenetic targets.

## Section 5: Epigenetic Relevant Chemical Space of Natural Products: Focus on Dnmt Inhibitors

In drug discovery it is generally accepted that a major benefit of natural products vs. purely synthetic organic molecules is, overall, the feasibility of the former to exert a biological activity and increased chemical diversity (Ho et al., 2018). The chemical space of natural products is vast and its molecular diversity has been quantified over the past few years (López-Vallejo et al., 2012; Olmedo et al., 2017; Shang et al., 2018). A major contribution to these studies has been the increasing availability of natural products collections in the public domain (Medina-Franco, 2015). Examples of major compound collections are the Traditional Chinese Medicine (Chen, 2011), natural products from Brazil – NuBBE (Pilon et al., 2017), AfroDb (Ntie-Kang et al., 2013) or collections available for screening in a medium to high-throughput screening mode. The large importance of natural products in drug discovery has boosted the development of open access applications to mine these rich repositories. Few examples are ChemGPS-NP, TCMAnalyzer, and other resources described elsewhere (Rosen et al., 2009; Chen et al., 2017; Gonzalez-Medina et al., 2017; Liu et al., 2018).

**FIGURE 3:**
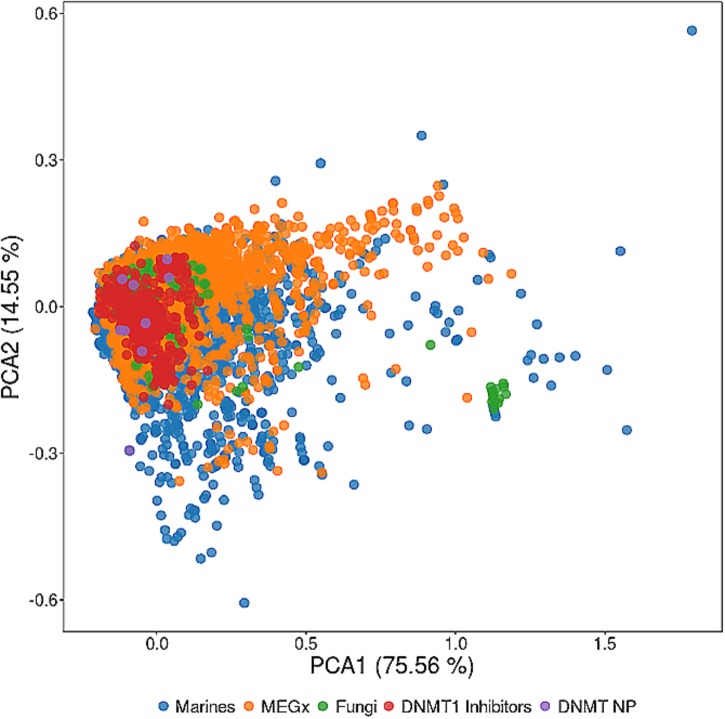
Visualization and comparison of the chemical space of DNMT inhibitors from natural sources (DNMT NP) vs. DNMT1 inhibitors and different natural products data sets. The visual representation of the chemical space was based on principal component analysis of six physicochemical properties of pharmaceutical interest. The percentage of variance is shown on each axis of the plot.

The chemical space of natural products from different sources has been compared to several other collections including the chemical space of drugs approved for clinical use and synthetic compounds (Olmedo et al., 2017; Shang et al., 2018). These studies demonstrate that the chemical space of natural products is vast, that there is a notable overlap with the chemical space of drugs, and that natural products cover novel regions of the chemical space. The overlap with the chemical space of approved drugs is not that surprising since there are a large percentage of drugs from natural origin. **Figure 3** shows a visual representation of the chemical space of 15 representative DNMT inhibitors from natural sources vs. 4103 compounds from a commercial vendor library of natural products, 206 fungi metabolites, and 6253 marine natural products (Krishna et al., 2017). The visual representation was generated with principal component analysis of six physicochemical properties of pharmaceutical relevance, namely molecular weight (MW), topological surface area (TPSA), number of hydrogen bond donors and acceptors (HBD/HBA), number of rotatable bonds (RB), and octanol/water partition coefficient (logP). The first two principal components capture about 90% of the total variance. The visual representation of the chemical space in this figure indicates that marine natural products (data points in blue) cover a broader area of the chemical space followed by natural products in the vendor collection (orange) and by fungi metabolites (green). DNMT inhibitors from natural origin (purple) are, in general, inside the subspace of the DNMT1 inhibitors (red). This visualization of the chemical space indicates that there would be expected to identify more DNMT1 inhibitors in the marine and vendor collections, as well as in the data set of fungi metabolites.

## Section 6: Opportunities for Searching for Natural Products as Dnmt Inhibitors

Most of the DNMT inhibitors from natural sources have been identified by serendipity. As discussed in Section 5, the chemical space of natural products and food chemicals can be explored in a systematic manner using computational approaches. A classical and general approach is using virtual screening. The main aim of virtual screening is filtering compound data sets to select a reduced number of compounds with increased probability to show biological activity. Virtual screening has proven to be useful to identify hit compounds (Clark, 2008; Lavecchia and Di Giovanni, 2013). **Table 1** summarizes representative case studies where virtual screening has led to the identification of active compounds with novel scaffolds. In other studies, virtual screening has uncovered potential active compounds but experimental validation still needs to be conducted. Examples of these studies are further discussed in the following sections.

**Table 1:** Summary of virtual screening hits as inhibitors of DNMTs.

Study	*In silico* approach	Major outcome	Reference
Structure-based screening of a lead-like subset of NP from ZINC	Cascade docking followed by a consensus approach	One computational had reported activity. Additional natural products were identified for screening.	Medina-Franco et al., 2011
Ligand- and structure-based screening of 800 NP	QSAR model based on linear discriminant analysis and consensus docking.	Six consensus hits were identified as potential inhibitors.	Maldonado-Rojas et al., 2015
Structure-based screening of 111,121 molecules.	Docking-based screening of synthetic screening compounds.	Identification of a low micromolar hit with a novel scaffold. Further similarity searching led to the identification of two more potent hits.	Chen et al., 2014
Ligand-based screening of 500 compounds.	Pharmacophore-based virtual screening.	Identification of one inhibitor of DNMT1 with activity in the low micromolar range. The hit showed some selectivity vs. DNMT3B.	Hassanzadeh et al., 2017
Structure- and ligand-based screening of 53,000 synthetic compounds.	Pharmacophore model, a Naïve Bayesian classification model, and ensemble docking.	Two compounds showed DNMT1 inhibitory activity at single but low concentration of 1 μM.	Krishna et al., 2017

*NP: natural products.*

There are several published studies of virtual screening of natural products to identify DNMT inhibitors and/or demethylating agents. In an early work, Medina-Franco et al. (2011) reported the screening of a lead-like subset of natural products available in ZINC. Authors of that work implemented a multistep virtual screening approach selecting consensus hits identified from three different docking programs. One computational hit showed DNMT1 activity in a previous study. Other candidate compounds were identified for later experimental validation (Medina-Franco et al., 2011).

In a separate work, Maldonado-Rojas et al. (2015) developed a QSAR model based on linear discriminant analysis to screen 800 natural products. Hits selected were further docked with two crystallographic structures of human DNMT employing two docking programs. Six consensus hits were identified as potential inhibitors (Maldonado-Rojas et al., 2015).

Virtual screening of synthetic libraries has also been reported to identify active compounds with novel scaffolds and suitable for lead optimization. For instance, Chen et al. (2014) reported a docking-based virtual screening of the commercial screening compound library SPECS with 111,121 molecules (after filtering compounds with undesirable physicochemical properties). Results of that work led to the identification of a compound with a novel scaffold with low micromolar IC_50_ (10.3 μM). Starting from the computational hit, similarity searching led to the identification of two more potent compounds.

Hassanzadeh et al. (2017) recently reported a pharmacophore-based virtual screening of a compound database with 500 compounds. The pharmacophore was generated using a ligand-based approach by superimposing a group of active nucleoside analogs. Selected hits, which are structurally related to the barbituric acid, were docked into the substrate binding site of DNMT1. One compound was identified with a novel chemical scaffold that inhibits DNMT1 in the low micromolar range (IC_50_ = 4.1 μM). The compound also showed some selectivity on DNMT1 over DNMT3 enzymes (Hassanzadeh et al., 2017).

Krishna et al. (2017) implemented a virtual screening protocol using several structure- and ligand-based approaches. Methods included a pharmacophore model, a Naïve Bayesian classification model, and ensemble docking. Three out of ten selected compounds from a commercial library of synthetic molecules (e.g., Maybridge with 53,000 small drug-like compounds), showed DNMT1 inhibitory activity at compound concentration of 20 μM. Two of these molecules showed activity at 1 μM (Krishna et al., 2017).

In addition to the studies discussed above and summarized in **Table 1**, the next subsections discuss other approaches that can be explored. Case studies for each strategy are outlined briefly.

### Section 6.1: Similarity-Based Virtual Screening of Natural Products

Similarity searching is a commonly used approach for identifying new hit compounds. Major goals are identifying starting points for later optimization or expand the SAR of analog series. Since similarity searching is fast it can be used to filter large chemical databases and it can be used in combination with other computational approaches such as molecular docking.

Similarity searching involves two major components: a molecular representation and a similarity coefficient. In practice, one of the most common molecular representations are two-dimensional (2D) fingerprints. A fingerprint is generally a string of zeros and ones that indicate the presence or absence of molecular features, respectively. In turn, one of the most common similarity coefficients is Tanimoto’s (Bajusz et al., 2015). Full discussion of molecular representations and similarity coefficients are published elsewhere (Willett et al., 1998; Maggiora et al., 2014).

A novel approach to encode the chemical structures of data sets is the database fingerprint (DBFP) (Fernández-de Gortari et al., 2017). The rationale of DBFP is account for the most structural features encoded in bit positions of an entire data set. In principle, virtually any data set can be represented. For instance, it can be a small or large chemical database of screening compounds or a group of active compounds. DBFP can be used in visual representation of the chemical space (Naveja and Medina-Franco, 2018) and similarity searching (Fernández-de Gortari et al., 2017). More recently, this approach was further refined into the so-called statistical based database fingerprint (SB-DFP). This approach has the same underlying idea and application of DBFP. A key improvement is the approach to account for the most relevant structural features that are derived from a statistical comparison between the structural features of a data set of interest vs. a database of reference.

### Section 6.2: Pharmacophore-Based

Thus far, several pharmacophore modeling studies have been conducted for inhibitors of DNMT1. Different approaches and input molecules have been used to develop these models. Most of the pharmacophore models have been employed to virtually screen chemical databases and identify novel hit compounds.

Yoo and Medina-Franco (2011) reported one of the first pharmacophore models for inhibitors of DNMT1. The model was generated based on the docking poses of 14 known inhibitors available at that time. The docking was conducted with a homology model of the catalytic domain of DNMT1. Of note, at the time of that study the crystallographic structure of human DNMT1 was not available. Known DNMT inhibitors used to develop the pharmacophore model included the natural products curcumin, parthenolide, EGCG and mahanine (Yoo and Medina-Franco, 2011). A year later was reported that trimethylaurintricarboxylic acid (**Figure 2**) showed a good agreement with this structure-based pharmacophore model. This compound is structurally related to 5,5^′^-methylenedisalicylic acid that has an inhibition of DNMT1 in a low micromolar range (IC_50_ = 4.79 μM) (Yoo and Medina-Franco, 2012; Yoo et al., 2012).

More recently, as described in the first part of Section 6, Hassanzadeh et al. (2017) developed a pharmacophore model based on a ligand-based approach by 3D superimposition of active nucleoside analogs. That model was used to do virtual screening (*vide supra*). In the same year, with the aid of the Hypogen module of the software DS4.1, Krishna et al. (2017) developed a ligand-based pharmacophore model using the structures of 20 compounds obtained from the literature. The model was validated with the classification of an external set with known active and inactive compounds. The validated pharmacophore models were employed as part of a combined strategy to identify novel active molecules (Krishna et al., 2017).

### Section 6.3: *De novo* Design

*De novo* design is a technique currently explored for DNMT inhibitors on a limited basis. Here we briefly outline two promising perspectives related to natural product research. The first one is a strategy that provides a structural diversity classification of natural products scaffolds through generative topographic map algorithm implementation often so-called chemographies. Chemographies allow the visualization of the landscape distribution of the chemical space of natural products and their synthetic mimetic compounds (Miyao et al., 2015). Since chemographies could be generated from pharmacophoric features and molecular descriptors, it would be feasible to do scaffold hopping based on the structures of natural products (Rodrigues et al., 2016). The second approach is based on scaffold simplification that could be adapted to generate fragment-like natural products focused on DNMT inhibitors. This strategy reduces the molecular framework of natural products through the implementation of a scaffold tree algorithm based on rule-based decomposition of ring systems (Bajorath, 2018).

## Section 7: Conclusion

Epigenetic targets are attractive to develop therapeutic strategies. DNA methyltransferases are the major enzyme family being one of the first epigenetic targets studied, in particular for the treatment of cancer. However, over the past few years, more therapeutic opportunities related to the modulation of DNMTs are emerging. Therefore, there is a growing interest in the scientific community to identify and develop small molecules that can be used as epi-drugs or epi-probes targeting DNMTs. Virtual screening has become more used in recent years to uncover natural products as inhibitors of DNMTs and/or demethylating agents. To this end, well stablished structure- and ligand-based virtual screening approaches are being used such as automated docking, QSAR and similarity searching. Also, novel chemoinformatic approaches are being developed. Of course, the computational methods should be validated with rigorous experiments *in vitro* and *in vivo* experiments to support their application.

Natural products have a well stablished history as inhibitors of DNMTs and demethylating molecules. However, most of the active natural products have been identified by serendipity. The knowledge of the three-dimensional structures of DNMTs in combination with increased *in silico* approaches and better computational resources are boosting the systematic search of bioactive molecules from natural origin. In addition, the increasing availability of natural product databases facilitates the discovery of epi-drugs and epi-probes targeting DNMTs.

## Section 8: Perspectives

Natural products inside or outside of the traditional drug-like chemical space represent a large promise to develop novel compounds with DNMT inhibitory activity or demethylating properties. This is because the traditional chemical space is highly represented by small molecules that over the past few years have not been very successful. A notable example in this direction is the reemergence of peptide-based drug discovery. Indeed, linear, cyclic peptides and peptidomimetics are regaining interest in drug discovery (Fosgerau and Hoffmann, 2015; Henninot et al., 2018).

Other promising an emerging avenue are the modulators of protein–protein interactions (PPIs) (Díaz-Eufracio et al., 2018). DNMTs are known to be involved in several PPIs (Díaz-Eufracio et al., 2018). Modulation of such interactions can be conveniently achieved with natural products. This is because PPIs are “difficult targets” not easily addressed by small molecules from the traditional chemical space (Villoutreix et al., 2014). In other words, since PPIs have unique features these can be approached with novel chemical libraries. Natural products collections represent excellent candidates for this purpose.

We foresee an augmented hit and led identification efforts based on natural products combining approaches such as high-throughput screening, structure-, ligand-based *in silico* screening, structure-based optimization, similarity searching, and scaffold hopping (Schneider et al., 1999). As part of the search for novel and more potent compounds is crucial to consider potential toxicity since toxicity issues play a major part in the lack of success of drug discovery projects.

## Disclaimer

A similar version of this manuscript was deposited in a Pre-Print server on July 6, 2018. The reference is: Saldívar-González, F. I.; Gómez-García, A.; Sánchez-Cruz, N.; Ruiz-Rios, J.; Pilón-Jiménez, B. A.; Medina-Franco, J. L. Computational Approaches to Identify Natural Products as Inhibitors of DNA Methyltransferases. *Preprints* 2018, 2018070116 (doi: 10.20944/preprints201807.0116.v1).

## Author Contributions

All authors contributed to methodology and formal analysis. FS-G, JR-R, and BP-J contributed to data curation. AG-G, FS-G, DC-PdL, and JM-F contributed to writing-original draft preparation. AG-G, FS-G, NS-G, and JM-F contributed to writing-review and editing. AG-G, FS-G, and BP-J contributed to visualization. JM-F contributed to project administration.

## Conflict of Interest Statement

The authors declare that the research was conducted in the absence of any commercial or financial relationships that could be construed as a potential conflict of interest.
